# Bibliographical Notices

**Published:** 1841-11-27

**Authors:** 


MEDICAL EXAMINER.

DEVOTED TO MEDICINE, SURGERY, AND THE COLLATERAL SCIENCES.

No. 48.] PHILADELPHIA, SATURDAY, NOVEMBER 27, 1841. [Vol. IV.

### BIBLIOGRAPHICAL NOTICES.

*Lecture, introductory to a Course on the Principles and Practice of Surgery in the University of Pennsylvania.* Delivered November, 1841. By William Gibson, M.D.

Professor Gibson's object, from beginning to end of this discourse, has been, he tells us, to speak for himself, well knowing that now-a-days every man must do that, or not be spoken of at all! Braving, then, or rather defying, the sarcasms which such self-glorification is apt to elicit, he has boldly undertaken the task of doing that justice to himself and his University, which he fears, amid the rivalry of conflicting interests, they may fail to receive at other hands. Presuming largely on the occasion of an introductory lecture, which we think fairly warrants the display of some amount of egotism, Dr. Gibson has furnished an outline of auto-biography, which, no doubt, was listened to with interest by the pupils assembled around him as the preceptor of their choice. This lecture Dr. Gibson has 'had much pleasure' in publishing, at the request of the class; and in publishing it he has at least weakened the force of the apology which he claimed in addressing it to his pupils. It is, however, a lively effusion, characterised by the sprightliness and vigour with which the Doctor generally writes. He was born, he informs us, some fifty years ago,—we suspected more,—in Baltimore; came to Philadelphia to study medicine, and heard his first lecture from his distinguished predecessor, Physick. Like other great men, Dr. Gibson had an early inkling of his future celebrity. Upon returning to his lodgings from this lecture of Physick's, he announced to his landlady his intention of one day occupying the professor's place. *En attendant*, Dr. G. went to Edinburgh where he graduated, after writing a thesis that

"Attracted the attention of reviewers, and was quoted by several scientific writers in England, but more especially by the Germans on the continent of Europe."

The Doctor afterwards became a private pupil of Sir Charles Bell, and, after remaining three years in all in Europe, returned to assist in getting up the University of Maryland.

"He was appointed to the chair of Surgery in that Institution; engaged extensively in private practice, medical as well as surgical, but chiefly the latter, and often made long journeys into Maryland, Virginia, and Pennsylvania, to perform important and difficult operations."

After a residence of nine years in Baltimore, upon the death of Dr. Dorsey, Dr. Gibson was called to the University of Pennsylvania. Dr. Physick abandoned the surgical chair to take the anatomical chair vacated by the death of Dr. Dorsey, and Dr. Gibson obtained the professorship of surgery. Almost immediately after removing to Philadelphia, Dr. Gibson was elected one of the surgeons to the Hospital of the Philadelphia Alms House, then situated in a central part of the city, but since removed to the western bank of the Schuylkill.

"For more than twenty years," says Dr. Gibson, "I have been the chief surgeon to the former and to the present hospital, have delivered an equal number of clinical courses, and during that time have performed as many capital operations as any hospital surgeon, perhaps, in the United States—owing to the hospital being more than twice the size of any other in the country, and to my having had always charge of the wards during the winter months, when the patients are most numerous and the cases most interesting. Within the last eighteen months, however, my colleagues and myself have consented to relinquish one half of our wards in favour of other schools of the city, not wishing to monopolize privileges which others for a long time have been anxious to enjoy conjointly with ourselves."

There are some inaccuracies in this statement. Dr. Gibson has never been *chief surgeon*, but one of four surgeons, to the hospital. It is, as he says, probably larger than any other in the country; but, since it has been removed to the buildings over the Schuylkill, it has never had the same amount of accidents and recent surgical cases that are to be found in the wards of the Pennsylvania Hospital. Some of the physicians and surgeons to the Philadelphia Hospital, it so happens, are professors in the University of Pennsylvania and the Jefferson Medical College, but we hardly think Dr. Gibson justified or politic in representing the hospital as an appendage of either or both of these institutions.

So much for the Professor's account of himself. We may add that he is a portly gentleman, of very pleasing address, a capital lecturer, gifted in a high degree with the ore rotundo, perspicuous and forcible in his language—but here is a portrait of 'an able and successful lecturer,' which, it may be suspected, was drawn from the original whom we are discussing. The natural qualities of such a lecturer are,

"An easy, quiet, and composed demeanour, a simplicity of thought and action devoid of all affectation, a manner free from embarrassment of every description, a clear and distinct enunciation, a voice sufficiently powerful to reach the most distant hearers, susceptible of modulation, marked by peculiar intonations, so regulated in its cadence as never to terminate abruptly, and with great capability of emphasis, whenever the necessity of such power may be called in requisition. If, to these natural attributes be joined good common sense, steadiness of purpose, a laudable ambition to excel, a natural love or turn for the subject to be taught, that will cause the bosom of the speaker to glow with enthusiasm and enable him to rouse and keep alive the attention of his hearers and fix it upon important points which he wishes to imprint indelibly upon the memory, while he descants soberly and quietly, and in the plainest possible style of elocution upon common place topics; if I repeat, to these natural qualifications there be added intimate acquaintance with the best authorities of the profession, ancient and modern, a profound knowledge of the subject, the result of experience or personal observation, associated with the power, rarely possessed, of separating the useful from the useless, of winnowing the chaff from the grain, joined to peculiar tact in presenting in the most palatable, but most solid and simple form every intellectual viand, garnished by the dainties that a liberal education can always throw around, such qualifications are sure to be followed by success; and will, in every possible way, richly deserve it."

Appended to the auto-biography, are some well conceived and expressed comments on the degrading pruriency of the day for surgical distinction through the medium of cutting and slashing. The importance of medical surgery is strongly urged, while operative surgery is to be deemed secondary and subordinate in its aim and application, not, however, that he undervalues the latter, holding, as he does,

"The highest respect and veneration for the man who, with a mind imbued with the profoundest knowledge of his profession, as shown by a general acquaintance with all its branches, can boldly and unerringly, and with matchless dexterity, plan and execute, successfully, operations which the mere professional mechanic would shrink from with apprehension and dismay, or be totally unable to comprehend; thereby demonstratiug that it is the *combination* of medical and operative talent tnat constitutes the prerogative of the great surgeon, and makes him a blessing to mankind."

Dr. Gibson does not forget to call attention to the "sixth edition of his Institutes of Surgery, just issued from the press," and makes an eloquent and just appeal in favour of the antiquity and long established reputation of the school with which he is connected. The Doctor, also, cautions diffident and nervous students against the mistaken idea that the examinations are so rigid in long established institutions as to render it very difficult for them to obtain a diploma. He assures them "that there will be no possibility of a pupil being rejected who is at all acquainted with his subject." These and other tempting promises we have no doubt the Doctor will amply fulfil.

---

*The Western and Southern Medical Recorder. Edited by* James Conquest Cross, M. D., *Professor of the Institutes of Medicine and Medical Jurisprudence in Transylvania University, &c.*

The first number (for the present month) of this Journal lies upon our table. It is an octavo of forty-eight pages, designed to appear on the first Monday of each month.

We welcome it as a medium of communication with the School of Transylvania, and the science of the highly cultivated city of Lexington.

---

*The Introductory Lecture to the Course of the Institutes of Medicine, in the University of Pennsylvania, for* 1841–2. By Samuel Jackson, M. D.

This is one of the most agreeable introductories we have met with, and adds not a little to Dr. Jackson's reputation as an eloquent and instructive lecturer.

The lecturer begins with the examination of the studies and mode of commencing necessary for a physician; after some introductory remarks, he illustrates the subject by a reference to a symptom—cough—sometimes of no value, expressing nothing but an irritation of a distant organ; sometimes a nervous cough, dedendent upon a general disorder of the nervous system, and sometimes a "protective, preservative" action, expelling injurious substances—whether introduced from the exterior, or formed by the organ itself,—or a strictly curative action, "to be respected in the treatment of the case."

Passing then to the study of the difficulties of medicine—as a mixture of art, science, and philosophy—a passing attention being given to the spirit and mode in which it was cultivated by the ancients, and the difficulties which continually obstructed it—we arrive at a new era, beginning with John Hunter, a great and original genius.

"John Hunter stood alone in that thick night, that had palled the science of medicine, for ages, in the dunnest cloud of ignorance. His genius, heaven-lighted, shone like a brilliant Pharos, throwing its illuminating rays into the surrounding gloom, giving a new direction, and animating to new exertions the long benighted wanderers of medical science. The whole face of the science soon changed. He introduced experiment into physiology, and at once constituted it a science. Every department of the physiological sciences was the object of his research, and felt the animating influence of his genius. He originated comparative anatomy and physiology, the cultivation of which is rapidly laying open the otherwise unapproachable mysteries of the human organization. Surgical and medical pathology, before entirely conjectural, assumed from his principles a more positive character, and to which could be fixed some specific ideas.

"It is true, that many things now well understood, John Hunter saw but dimly shadowed in the darkness which his genius was dispelling, unaided by other lights than his own. He laboured, too, with difficulty in attempting to express, in language, new phenomena, to convey to other minds the great conceptions with which his own was teeming, and that had never before entered the thoughts of man. His style is obscure. His expressions are not the most definite or precise. This is the carping criticism of little minds, incapable of estimating original men, *true men*, who delve in the depths of nature and penetrate the inmost recesses of thought."

Another mind, which has produced nearly as powerful, and perhaps a more extended influence, is that of Bichat, whose life was so early sacrificed to his unremitting labours:

"Bichat may be considered the principal originator of positive science in the medicine of France. He did not, like Hunter, stand alone. Bordeu and Pinel had preceded him, and the illumination of their minds, had already dissipated the first difficulties of his path.

"The genius of Bichat was active, brilliant, and vigorous; but it did not possess the depth, and energy, and extent of that of Hunter.—The light that emanated from Bichat's intellect, was concentrated on a few objects and thrown in one direction. Those objects were seen distinctly, and the impression they imparted was vivid and exciting.

"Hunter's mind irradiated far, and wide, and in every direction, the deep obscurity of the science: but often the things discovered were not seen in a full and striking outline, nor their connections clearly displayed.

"With less power, Bichat, probably, imparted a more vigorous impulse to the scientific movement of medicine, than Hunter, with his greater depth and profounder knowledge."

Haller is also mentioned as one of the most eminently practical and laborious cultivators of medicine, looking far beyond his age, and obstructed rather by the imperfect condition of the collateral sciences than from want of labour or deep searching sagacity.

Returning again to the oft-quoted and admired Hippocratic axiom, which is the introductory sentence of the lecture, *Ars longa, vita brevis*, Dr. Jackson inquires into the present state of medicine, forming a picture, correct, indeed, in most respects, but by far too discouraging, of the pretensions of medicine in its actual state to the title of a philosophical science. It is undoubtedly true that it is still most imperfect, in a state of transition, destined to approach much nearer to demonstrative truth, and to admit of much more correct and just relation of the phenomena of disease and the remedies which we are able to apply; but still a multitude of leading facts are known and settled; and, although our remedial means too often fail us, our powers of prediction and discrimination are not often at fault: much is settled and positive, much will undoubtedly become so, and something will in all probability ever remain obscure and incapable of sure analysis, for we are living, feeling beings, and the rigour of calculation which is possible in the relations of the inorganic world, must often be baffled by the numerous controlling influences to which the human body and the human mind are exposed.

"At the present period, the utmost activity pervades the science of medicine. It rapidly hastens to its completion. No department is left unexplored. Facts are rapidly developed, tested, and established by positive and reiterted observations and experiments. It is not a single though a glorious intellect that brightens, for a space, a darkened region; but from every nook and corner, and "coin of vantage," stream out gleams of light, whose aggregate illumination lighten up every recess and department with the brilliancy of day.

"More than three thousand years have witnessed the progress of medicine. Yet, you perceive, Gentlemen! that it has not been perfected, as a science, indispensable to its completion as an art. How just, then, the axiom which heads this discourse—*Ars longa.*

"If medicine, as a science, from the nnmber and diversity of its phenomena, has not yet reached its fulfilment, the time is still distant when it may be approached as a philosophy. As science is necessarily preceded by art, so philosophy must follow on the cultivation of science. In the present state of the science, the philosophy of medicine must be limited to cautious generalization of its principles.

"Art, Science, and Philosophy have been spoken of as distinct departments of knowledge. That our meaning may be more clear, it will be proper to exhibit their differences.

"Art is knowledge acquired by experience. Its facts are simply historical, unconnected with their causes or the laws that compel their existence. The facts themselves are applied in practice, without an understanding of their operation. The practice of the art is then empirical.

"Science is the knowledge of phenomena, in their relations of cause and effect, and the determination of the fixed laws that govern them. Nothing in the creation is a random result. Every thing has been foreseen and provided for. Every phenomenon occurs in the order and connexion intended for it. Some phenomena are constant, the series to which they belong are always in action, and they are regarded as immutable.

"Thus, respiration of atmospheric air, circulation of blood, development of nervous power, vital activity, are an immutable series of phenomena. One cannot change without all being affected; one cannot exist without, or independent of the other. They establish a law of life. But all the physiological phenomena are equally permanent: they all result from equally established laws, necessarily productive of each phenomenon in its appropriate series, and in its position in that series. If the natural conditions are all present, for any organ, its functions or phenomenal acts must exist. The stomach must digest, a gland secrete, a sensory nerve feel, a motor nerve excite, a muscle contract, a sense give perception. The law is positive: the effect must ensue, when the cause is present.

"But the same positiveness attends on the occasional, or contingent phenomena, as they are manifested in disease. An inflammation, a fever, a palsy, a spasm, an abscess, vomiting, purging, any possible modification of the state of an organ, or its function, as they may occur singly or in combination, are not less the result of laws provided for their occurrence.

"They are parts of series, or formulas of phenomena;—have each their cause preceding them, as they are the causes of what necessarily follows them. There can arise no possible combination of the numerous elements, that compose the living organism of man, many of which have, probably, not yet taken place, but the law already exists, that determines the phenomena that do, or that will result.

"Science then consists in the ascertainment of the existence and nature of phenomena, the arrangement of them in their specific categories, and the invariable order of their connexion. When this has been accomplished, a law of nature has been discovered, immutable in the production of the phenomena that obey it.

"Science can be predicated of no scheme of knowledge, if the phenomena of which it treats, are not connected in a chain of dependant facts, arranged in the order of their connexion.—When this has been effected, science is finished, and is positive. The possessor of it, has the power, any fact being given, to assert all that had preceded that fact. He traces the filiation of its anteceding phenomena to the primary or fundamental cause; and he can predict all that will ensue, from knowing the links that depend upon it. But a knowledge of the laws of nature, enables us to control them, to modify and bend them to our purposes. Science arms us with power; and the man of science is more efficient, yet safer, beyond calculation, over the mere man of art, or yet ruder empiric.

"Philosophy is unconnected with the research into causes and effects. Phenomena, the object of the senses, and the subject of science, belong not to philosophy. It is the development of general truths by the operation of the mind, from pure reason; the establishment of fundamental and primary facts, the governing principles of the phenomena of the universe, all combined in one unity of plan, emanating from the divine creative intelligence, that compose the great questions of philosophy. These are subjects not cognizable by the senses; that are beyond the grasp of the understanding, that exist independent of matter or phenomena: they belong to the divine idea, and can be comprehended only by that feeble emanation of the divine intelligence, imparted to man in his Pure Reason.

"In the natural order of acquiring knowledge, were human knowledge once perfected, the commencement would be the study of philosophy, or the reason for the existence of phenomena, and the laws they obey: the second step would be the knowledge of science, or of the laws governing and arranging the production of the phenomena; and, finally, the last subject of investigation would be the phenomena themselves, and the application of knowledge to accomplish results.

"From this view, it is fairly apparent, that medicine is yet imperfect as an art, incomplete as a science, and almost wanting in philosophy. Again we find most true is the assertion: *Ars longa est.*"

The difficulties of the science, in the abstract, are increased by the imperfect power of each individual to master it in its present condition; hence, too, many content themselves with the mere practice of the art; and some, degrading it much farther, look upon it only as a trade by which they are to gain their bread, without ever reaching the complex but yet established rules of art. In fact, there is no complete divorce between the art and the science, the former cannot be well understood without a knowledge of the latter, and he who would remain a mere workman degenerates into an empiric. Another, and still a numerous class, rely upon a false art, much inferior in morals to the true but imperfect therapeutics of medicine, put forth false pretences to cures, or embrace some false system which may for a time enjoy a passing notoriety. How much may be done by each individual, and how much labour is required and is due from every aspirant to the profession of medicine, is fairly set forth.

"The second point of view, in which it was proposed to regard the length of art, contrasted with the shortness of life,—*vita brevis*—was, in reference to the individual capacity, in becoming perfected in the art of medicine.

The expansion given to medicine by its cultivation as a science, the creation of many new departments of research, the acknowledged supremacy of science over mere art, undoubtedly render a complete knowledge of the medicine of this period a work of immense labour, but of proportionate value. It can be accomplished only by application and perseverance, conjoined to strong natural abilities. Velpeau is a living example of how much can be done, and well done, by one who is determined to accomplish all that lies within the reach of his capacity.

From the difficulties that attend an extended knowledge of medicine, there is a strong tendency manifested to cultivate the specialities of the science. Some one branch of investigation, some department of the profession, or some one class of diseases, is selected, to which there is an exclusive devotion of time and application.

To a certain extent this method possesses some advantages. It must not be carried too far. A speciality cannot be detached from the science: it is a part of it, and can be properly understood and treated, especially the exceptional or irregular cases that must arise at some time, only when the general principles of the science can be applied to the explanation of its phenomena, whether regular or anomalous.

There is another source that adds to the difficulties encumbering medicine, as an art, and increases the obstacles to the being perfected in its practice. This source is, in addition to the number of pathological affections and their attending symptoms, the incessant revolutions that characterize diseases. No fact is better substantiated than that diseases, from some unknown laws that influence the vitality of living beings, especially man, present themselves in constantly varying forms. This instability of disease baffles experience. At one period, there prevails a uniform inflammatory tendency, and the anti-phlogistic and evacuant methods of treatment are more or less applicable to most cases of disease that occur during the ascendency of that constitution.

Soon after follows another constitution the reverse of this. All diseases take on a low or typhoid type,—the adynamic and ataxic states of disease,—and a sustaining method is demanded.

At another period, malarial diseases spread over extensive districts, filling every habitation with protean forms of febrile disease,—simple and malignant intermittents, pseudo remittents, and anomalous nervous disorders,—for which the great remedy cinchona or its alkaloids, must be relied on as the basis of a treatment.

Other prevailing forms might be enumerated, but these will suffice to establish the position we have laid down.

Intercurrent with these universal epidemic constitutions, occur especial epidemics; as small pox, measles, scarlet fever, influenza; all of which are modified, in each period of their return, by the prevailing constitution, and require a corresponding modification of treatment.

The epidemic constitutions that have been designated, succeed in cyles varying in duration from ten to twenty years, with intermediate cyles, marked by no particular influence.

From this fluctuating movement of disease, the practice of medicine never can be settled down on established formulas, determined remedies and doses. A physician should never harness himself to any doctrine or to any practice. The experience, tact and routine, he has acquired in one cycle suddenly fail him when a new one sets in. His studies, observations and experience, in treating disease, must recommence again for each succeeding cycle of disease, as it circles in the course of time. The shortness of life,—vita brevis—scarcely admits of perfectness in an art, at once complicated and changeable, without zeal, assiduity and devotedness to the acquisition of knowledge.

This picture of the difficulties of your professional life is no exaggeration. It is not intended, in presenting them to you at this time, to discourage you, but to prepare you to expect,—to encounter,—to vanquish them.

Half of the difficulties that necessarily beset us in life disappear when we know what they are. The means to avoid, or when avoidable, to overcome them, become apparent.

It is evident, from the nature of the difficulties that belong to medicine, as it is now constituted, that they arise from the paucity, or want of fixed principles, and of the ascertained laws of life, which would furnish an explanation of all its phenomena, and a clue to the means of governing them, as far as they are susceptible of control by human means.

Although the principles and laws of the whole science are incomplete, yet many are fairly established, and applicable with success to the solution of perplexing symptoms, and the treatment of intricate cases.

In your studies, then, endeavour to lay hold of the fundamental, or primary phenomena of the animal organism. From these commence, and consecutively follow all the phenomena that you are called on to observe and comprehend.

When you possess these, you have the compass and the chart that will guide you in safety, though your way be trackless. You must take care, however, that you mistake not secondary for fundamental phenomena, and assumptions for truths,—the wreck of so many ambitious hopes, the cause of so much self-deception!

There are fundamental truths in medicine. They are known by their perennity. They have never been entirely lost to the science, even in its gloom and depression, but have always re-appeared, whenever an original mind has applied its forces to the investigation of phenomena, and sought in the recesses of its reason for the laws of their existence.

The most important of these, on which medical science must ever repose, and must always form the basis of a sound practice, I will now present to you, as the commencement and the ending of medical investigations.

As these principles are present or absent, in any doctrine of medicine, will it be a truth or a fallacy.

These primary or fundamental facts of the first consequence, may be arranged in the following series:

1. Life is a result of an unknown force, consonant to all the forces of nature; entering with them in the unity of plan on which the Creator has constructed the universe. It is called the vital principle, or organic force.

2. This principle or force is, consequently, in relation with all the other forces of the universe: is influeuced by, acts on, and re-acts with them.

3. Its activity is exerted exclusively on organic matter, and is displayed alone in animated organisms, or living beings, vegetable and animal.

4. The essential character of its mode of activity is movement: the cessation of movement is death: its activity is excited and maintained by the forces of external and internal agents.

5. The direct phenomena of life are effects or results of the actions and re-actions occurring between the vital principle or organic force, external forces, and organic matter. In the action of this triad, the organic force is the primordial principle of life; organic matter, in a fluid state, the primary element of life; external forces—caloric, electricity, oxygen—the primary exciters of life: organization is the result or product of vital activity.

The secondary phenomena of life, are the functions or offices of the organs. The organs are the instruments, the functions are the means of life, in the higher animals. The functions produce and maintain the indispensable conditions of the primary vital phenomena, while they are, at the same time, dependent on the primary phenomena for their existence.—*Abuentes in cirrulo.*

The functions form two distinct classes. The one class, purely chemical and mechanical in their nature, produce and maintain the organic and physical conditions of life. They are, the digestive or alimentary functions, respiration, circulation, secretion. They constitute the functions of organic or vegetative life, in the works of Physiologists.

The second class comprise the sensory and motor powers of the cerebro-spinal axis, and the contractility of the muscular fibres—irritability of Haller. They are the vital forces of Haller and others; but are clearly nervous functions. They are Dynamic functions, and their forces are the causes of the actions of the first class. They are known as the functions of animal life in systematic works.

7. The organic or vital force, in the production of phenomena, exhibits the following especial orders of phenomena:

*a.* It is creative: From a formless fluid organic matter—albuminous in animals, gummous in vegetables—there is developed, as the first result of its activity, germinal points, or nucleoli and nuclei; from which proceed germinative cysts, from which again are produced tissues, organs, living beings: the whole and each part constructed from an ideal type, existing in the eternal and creative intelligence of God.

*b.* It is conservative: The organization of living beings is never persistent. Every atom of every tissue is dying and re-produced at each moment of time. Life and death are inseparable and necessary relations.

But the form, the composition, structure, properties of each tissue and organ are preserved and re-produced always the same, under the same circumstances. The conservation of the natural condition, is the prolongation of the creative action of life.

*c.* It is medicative or therapeutic: The re-actions producing vital phenomena are subject to be disturbed by the action of numerous exterior forces, that are not in relation or harmony with the vital principle or organic force, either of the whole organism in its unity, or in some of its special manifestations in particular tissues and organs. This perturbed condition of the physiological re-actions of life, would necessarily end in the destruction of the natural organization, by perverting or suspending its formative and conservative operations. But provision is made to guard the economy from those destroying influences. The re-actions excited in the organs, or general organism, and the disorder of functions, named symptoms, are, for the most part, protective or recuperative processes, by which the economy is rescued from impending mischief and danger.

Vomiting, purging, expectoration, sweating, diuresis, a hæmorrhage, an inflammation, fever, even spasm and convulsions, are pathological functions that expel offending causes, depurate the vital fluid, or dispel a pathological condition that has taken root in some tissue or organ, and whose function is injured or destroyed.

The disease belongs essentially to the modification of the vital activity, disordered and perverted in its mode of action. The symptoms of the disease, which we name, and too often regard as essentially the disease, are, in reality, the defensive and curative operations of the economy. Let us be on our guard that this false view do not betray us into dangerous interference with that which is salutary in its intention.

The physician, in his true character, is the minister, the interpreter, the adjuvant of nature; not her master. He guides, controls, follows, obeys as she dictates and requires; or he wisely suffers her to complete her own work, when the laws and means she operates by, are adequate to acomplish the end.

There is one more fundamental principle, to which I would direct your attention.

Diseases once established, have a regular course to run, and observe laws for their recovery. All reactive diseases must, in a definite time, come to a conclusion. They must end in recovery, or death, or a chronic disease of the affected organ, most generally the consequence of a change in its structure.

When the constitution is good, and the disease not so powerful as to break down the vital forces of the organs, a recovery must ensue. These spontaneous cures are the boasts of the pretender; and, to the ignorant, the evidence of skill.

There can be no doubt as to the good tendency of lectures conceived in this earnest and elevated strain. They form the mind and character of the pupil, and inspire him with the enthusiastic devotion to his science which is at last the only real guide to honest, legitimate success.

